# Modulation of Hypoxia-Induced Chemoresistance to Polymeric Micellar Cisplatin: The Effect of Ligand Modification of Micellar Carrier Versus Inhibition of the Mediators of Drug Resistance

**DOI:** 10.3390/pharmaceutics10040196

**Published:** 2018-10-21

**Authors:** Hoda Soleymani Abyaneh, Amir Hassan Soleimani, Mohammad Reza Vakili, Rania Soudy, Kamaljit Kaur, Francesco Cuda, Ali Tavassoli, Afsaneh Lavasanifar

**Affiliations:** 1 Faculty of Pharmacy and Pharmaceutical Sciences, University of Alberta, Edmonton, AB T6G 2E1, Canada; hoda1@ualberta.ca (H.S.A.); asoleimani212@gmail.com (A.H.S.); vakili@ualberta.ca (M.R.V.); soudy@ualberta.ca (R.S.); kkaur@chapman.edu (K.K.); 2 Faculty of Pharmacy, Cairo University, Kasr El-Aini, Cairo 11562, Egypt; 3 School of Pharmacy, Chapman University, Irvine, CA 92618, USA; 4 School of Chemistry, University of Southampton, Southampton SO17 1BJ, UK; francesco_cuda@yahoo.co.uk (F.C.); A.Tavassoli@soton.ac.uk (A.T.); 5 Department of Chemical & Materials Engineering, Faculty of Engineering, University of Alberta, Edmonton, AB T6G 1H9, Canada

**Keywords:** hypoxia-induced chemoresistance, cisplatin, polymeric micelle, EGFR-targeted therapy, STAT3, HIF-1, GE11 peptide, pharmacological Inhibitors of HIF-1 and STAT3, combination therapy

## Abstract

Hypoxia can induce chemoresistance, which is a significant clinical obstacle in cancer therapy. Here, we assessed development of hypoxia-induced chemoresistance (HICR) against free versus polymeric cisplatin micelles in a triple negative breast cancer cell line, MDA-MB-231. We then explored two strategies for the modulation of HICR against cisplatin micelles: a) the development of actively targeted micelles; and b) combination therapy with modulators of HICR in MDA-MB-231 cells. Actively targeted cisplatin micelles were prepared through surface modification of acetal-poly(ethylene oxide)-poly(α-carboxyl-ε-caprolactone) (acetal-PEO-PCCL) micelles with epidermal growth factor receptor (EGFR)-targeting peptide, GE11 (YHWYGYTPQNVI). Our results showed that hypoxia induced resistance against free and cisplatin micelles in MDA-MB-231 cells. A significant increase in micellar cisplatin uptake was observed in MDA-MB-231 cells that overexpress EGFR, following surface modification of micelles with GE11. This did not lead to increased cytotoxicity of micellar cisplatin, however. On the other hand, the addition of pharmacological inhibitors of key molecules involved in HICR in MDA-MB-231 cells, i.e., inhibitors of hypoxia inducing factor-1 (HIF-1) and signal transducer and activator of transcription 3 (STAT3), substantially enhanced the cytotoxicity of free and cisplatin micelles. The results indicated the potential benefit of combination therapy with HIF-1 and STAT3 inhibitors in overcoming HICR to free or micellar cisplatin.

## 1. Introduction

Hypoxia is a common feature of solid tumors. Hypoxic areas in tumors are defined as regions with lower oxygen (O_2_) levels than physiological oxygen concentrations [1]. Hypoxia arises when the need for oxygen exceeds its supply. Hypoxia-induced chemoresistance (HICR) has been observed in a number of human cancers, including triple negative breast cancer (TNBC) [2,3,4,5], the most deadly and therapy-resistant type of breast cancer [6]. The development of HICR in TNBC is a significant clinical obstacle against effective cancer therapy. This necessitates the development of new strategies that can prevent or overcome HICR in TNBC.

Cisplatin is a platinum drug used as part of a standard chemotherapy regimen in TNBC patients [7,8]. However, its use in cancer patients leads to the emergence of severe side effects including renal damage, deafness, and peripheral neuropathy. The use of nanocarriers has been extensively studied in recent decades as a means to attenuate the toxic side effects of anticancer agents. Several different nanocarriers of cisplatin have been reported in the literature, from which a few have found their way to clinical trials [9]. The developed nano-formulations have been mostly effective in lowering the side effects of cisplatin in preclinical models. However, they failed to potentiate the anticancer effects of drug [10,11].

Reports on the effect of nano-delivery of anticancer drugs on HICR are limited. This is of particular interest, as delivery of anticancer drugs by their nano-formulations may, in fact, restrict drug access and movement to hypoxic regions of the tumor where resistant cancer cells may be present. To circumvent the problem of nano-formulation penetration to hypoxic regions of solid tumors, incorporation of nanoparticles in non-malignant cells that display inherent hypoxia-targeting abilities, such as monocytes, macrophages or even neural stem cells [12,13], or modification of the surface of nanoparticles by tumor-penetrating peptides has been tried by different research groups [14]. These strategies sought to enhance the penetration of nano-drug delivery systems at the solid tumor at a tissue level.

This study aimed to study the effect of cisplatin delivery by a stealth nano-formulation on HICR at a cellular level and explore strategies to circumvent HICR. For this purpose, we used a previously reported polymeric micellar formulation for cisplatin developed by our group [15] and assessed the cytotoxicity of cisplatin as part of this formulation versus free drug in normoxic versus hypoxic MDA-MB-231 cells. We then explored two strategies for the modulation of hypoxia-induced cisplatin resistance in the same cell line; first, by active targeting of cisplatin micelles to MDA-MB-231 cells, to enhance intracellular cisplatin levels, and second, by combining cisplatin or its nano-formulations with modulators of HICR in MDA-MB-231 cells.

For the purpose of active drug targeting, we chose modification of polymeric micellar cisplatin with peptide ligands against epidermal growth factor receptor (EGFR), since coexistence of hypoxia and high levels of EGFR expression is a known feature of TNBC [16,17]. We postulated the high expression of EGFR on the hypoxic TNBC cells can be exploited to achieve enhanced delivery of cancer therapeutics to the cells [17,18].

Targeted nanocarriers can significantly improve drug performance by delivering a high payload of drug to cancer cells [19,20,21,22]. Previous studies have provided support for the use of EGFR monoclonal antibody for the development of ligand guided nanocarriers for the purpose of tumor imaging or targeted drug delivery [23,24,25,26,27]. The high molecular weight of the full-length antibody, however, may compromise the penetration of antibody modified nanocarriers into tumor tissue, particularly in hypoxic tumor regions. It may also enhance the chance of nanocarrier removal by the reticuloendothelial system following opsonization in the blood circulation [28]. Furthermore, the high cost of the full-length antibody and limitations on the use of organic solvents for its conjugation to the surface of nanocarriers prohibits the wide use of antibodies as ligands for tumor-targeted nanocarriers. In this context, use of an EGFR-specific peptide, GE11, may be a better option. GE11 is a 12-residue peptide (YHWYGYTPQNVI) which was originally developed using phage display technique [29]. GE11 peptide shows a lower affinity for EGFR than its natural ligand, EGF, however, it provides the advantage of lower mitogenic activity. Overall, due to its high EGFR affinity, minimum immunogenicity, and relatively cheap method of synthesis and scale-up, GE11 has been widely conjugated to a variety of nanocarriers, including liposomes, polymeric micelles, as well as gold and gelatin nanoparticles [28,29].

In this study, we developed GE11 modified polymeric micellar complexes of cisplatin and assessed the success of this approach in enhancing cellular delivery of cisplatin and overcoming HICR to cisplatin in a TNBC associated cell line, i.e., MDA-MB-231 cells. We then investigated the effect of adding pharmacological inhibitors of hypoxia inducing factor-1 (HIF-1) and signal transducer and activator of transcription 3 (STAT3), as the key modulators of HICR in this cell line [4,30], on anticancer activity of polymeric micellar formulations of cisplatin versus free drug.

## 2. Materials and Methods

### 2.1. Materials

Cisplatin (cis-diamminedichloroplatinum(II) (CDDP) (purity 99%), #H878, was purchased from, AK Scientific Inc., Union City, CA, USA. Methoxy-PEO 5000 (MePEO), sodium cyanoborohydride, and Stattic were obtained from Sigma, St Louis, MO, USA. Stannous octoate was dried and purified using anhydrous magnesium sulfate, dry toluene, and vacuum distillation [31]. α-Benzylcarboxylate-ε-caprolactone (BCL) was synthesized by Alberta Research Chemicals Inc. (ARCI, Edmonton, AB, Canada) based on methods published previously by our group [32]. All other chemicals and reagents used were of analytical grade.

#### 2.1.1. Synthesis of Block Copolymers with Functionalized Poly(ethylene oxide) (PEO)

Acetal-poly(ethylene oxide) (acetal-PEO) was synthesized based on the previously published method by Nagasaki et al. [33]. Briefly, potassium naphthalene, the catalyst, was freshly prepared before the synthesis of acetal-PEO. To prepare the catalyst, 1.65 g (12.9 mmol) naphthalene and 0.575 g (14.7 mmol) potassium were added into 50 mL anhydrous tetrahydrofuran (THF). The reaction was protected under dry argon gas and kept running for 24 h, until a dark green color was obtained. To prepare acetal-PEO, 0.3 mL (2 mmol) 3,3-diethoxy propanol, the initiator, was first added into 40 mL anhydrous THF in a three-neck round bottom flask. The flask was purged with dry argon gas and maintained under an argon atmosphere. The catalyst solution (7 mL, ∼2 mmol) was added dropwise into the reaction solution to activate the initiator. After 10 min of stirring, the flask was transferred into an ice water bath. Ethylene oxide (11.4 mL, 228 mmol) was added into the reaction solution. After 48 h, the reaction was quenched by acidified ethanol (2 mL). Acetal-PEO was recovered by precipitation in ethyl ether. The product was purified by dissolution in THF and precipitation in ethyl ether, and vacuum dried for further use.

Synthesis of acetal-PEO-poly-(ε-caprolactone) (acetal-PEO-PCL) and acetal-PEO-poly(α-benzyl carboxylate-ε-caprolactone) (acetal-PEO-PBCL) block copolymers has been described in our previous publications in detail [34]. Acetal-PEO-PBCL was first prepared through ring opening bulk polymerization of BCL with acetal-PEO as an initiator. This was followed by hydrogen reduction of PBCL block catalyzed by Pd/charcoal. Briefly, 600 mg (0.1 mmol) acetal-PEO was reacted with 500 mg (2 mmol) BCL under vacuum at 145 °C for 6 h using stannous octoate as catalyst. Then the benzyl groups on acetal-PEO-PBCL were removed through hydrogen reduction in anhydrous THF catalyzed by Pd/charcoal. The produced acetal-PEO-PCCL was recovered and purified by precipitation in hexane.

#### 2.1.2. Synthesis of GE11 Peptide and GE11 Conjugation to Poly(ethylene oxide)-poly(α-carboxyl-ε-caprolactone) (PEO-PCCL) Block Copolymers

The GE11 peptide (NH_2_-YHWYGYTPQNVI-COOH) (Figure S1a) was synthesized chemically using standard Fmoc solid phase peptide synthesis as described previously by the laboratory of Kaur et al. [35]. Briefly, the first amino acid, isoleucine, was coupled to a 2-chlorotrityl resin (0.1 mM) (NovaBiochem, San Diego, CA, USA) at 5-fold excess using the N,N diisopropyl ethylamine (DIPEA) at room temperature for 5 h. Further amino acids were added automatically using an automated peptide synthesizer (Tribute, Protein Technology, Inc., Tucson, AZ, USA). The completed peptide was ultimately released from the resin with a mixture of 90% trifluoroacetic acid (TFA), 9% dichloromethane, and 1% triisopropylsilane (∼10 mL) for 90 min at room temperature. The cleaved peptide combined with TFA was then concentrated, washed with diethyl ether, dissolved in water and purified. Purification was done using C18 semi-preparative (1 cm × 25 cm, 5 μm) reverse-phase high-pressure liquid chromatography (HPLC) (Varian Prostar, MD, USA) with a gradient of acetonitrile−H_2_O (10−70% containing 0.05% TFA, 2 mL/min, 45 min run time). The peptide solution was freeze-dried to give pure peptide as a white powder. Analytical (0.46 cm × 25 cm, 5 μm) HPLC revealed a purity of 97% at 220 nm with retention time (Rt) = 13 min, and the MALDITOF (Voyager spectrometer, Applied Biosystems, Foster City, CA, USA) mass analysis showed [M + H]^+^ for the peptide as 1541.6 (calculated 1540.7) (Figure S1b).

The GE11 peptide was conjugated to the micellar surface through a reaction with the functional acetal groups on the micellar shell [34]. First, acetal-PEO_6000_-PCCL_3000_ (with 87% reduction of PBCL to PCCL) was assembled into polymeric micelles. Briefly, a diblock copolymer of acetal-PEO_6000_-PCCL_3000_ (20 mg) was dissolved in 1 mL acetone and added dropwise to 4 mL water while stirring. The solution was stirred for 24 h under a fume hood to remove acetone by evaporation. On the following day, the aqueous solution of polymeric micelles was acidified to pH 2 with 0.5 M HCl and stirred for 1 h at room temperature to produce aldehyde modified polymeric micelles. The resulting solution was then neutralized with 0.5 M NaOH. The osmolarity of the micellar solution was adjusted by addition of an appropriate volume of concentrated 10X P phosphate-buffered saline (PBS). An aqueous solution of the peptide (1.95 mg peptide in 500 µL of 1% dimethyl sulfoxide (DMSO)) (1:2 peptide to polymer, mole:mole ratio) was added and incubated with the aldehyde bearing micelles at room temperature for 2 h under moderate stirring. Subsequently, sodium cyanoborohydride (NaBH_3_CN) (1 mg) was added to the polymer to reduce the Schiff’s base. After 48 h of reaction, the unreacted peptide and reducing agent were removed by extensive dialysis using Spectrapor, MWCO 3500 (Spectrum Laboratories, Inc., Rancho Dominguez, CA, USA) against distilled water (24 h). The conjugation efficiency of the peptide to polymeric micelle was assessed by gradient reversed phase HPLC method measuring unreacted peptide concentration. A µ Bondpack (Waters Corp., Milford, MA, USA) C18 analytical column (10 µm 3.9 × 300 mm) was used. Gradient elution was performed at a flow rate of 1 mL/min using a Varian Prostar 210 HPLC System. Detection was performed at 214 nm using a Varian 335 detector (Varian Inc., Mulgrave, Australia). The mobile phase consisted of 0.1% TFA in H_2_O (solution A) and acetonitrile (solution B). The mobile phase was programmed as follows: (1) 100% A for 1 min, (2) linear gradient from 100% A to 60% A in 20 min, (3) linear gradient from 60% A to 0% A in 4 min, (4) 0% A for 2 min, (5) linear gradient from 0% A to 100% A in 4 min, and (6) 100% A for 5 min. The concentration of unreacted peptide was calculated based on a calibration curve for the peak height of known concentrations of GE11 peptide in aqueous solution of 1% DMSO. The amount of conjugated peptide was calculated by subtracting the amount of unreacted peptide from the initial peptide added to the reaction. The peptide conjugated polymer was then freeze-dried until further use. ^1^H NMR was performed on a Bruker, ASENDTM 600 MHz spectrometer (Billerica, MA, USA) to confirm the conjugation of the GE11 peptide on PEO-PCCL block polymer. Samples (GE11 peptide, acetal-PEO-PCCL, and GE11-PEO-PCCL) were dissolved in deuterated DMSO at a concentration range of 3–5 mg/mL and ^1^H NMR spectra were generated.

#### 2.1.3. Preparation of Plain and GE11 Cisplatin Micelles

GE11-PEO-PCCL or PEO-PCCL block copolymers were assembled into cisplatin polymeric micelles as reported previously with slight modification [15]. Briefly, either of the diblock copolymer (20 mg) was mixed with 4 mL aqueous solution of cisplatin (20 mg) and sodium bicarbonate (4–5 mg). The mixture was stirred for 24 h at room temperature. We couldn’t dissolve the whole amount of cisplatin in such volume, but high cisplatin levels were identified to be required to enhance complexation with the polymer and increase its loading levels. After micelle preparation, initially using centrifugation, the undissolved portion of cisplatin was separated from the micellar solution. In the next step, the unbound cisplatin was removed by ultrafiltration (3600× *g* for 40 min) using Centricon^®^ plus centrifugal filter units (MWCO 3 KDa, Millipore, Billerica, MA, USA) and micelles were re-suspend in 4 mL doubly distilled water. The final concertation of cisplatin was determined using ion coupled plasma mass spectrometer (ICP-MS).

#### 2.1.4. Measurement of the Size and Zeta Potential of Plain and GE11 Cisplatin Micelles

The average hydrodynamic diameter and size distribution of the GE11 cisplatin micelles were estimated and compared to plain cisplatin micelles by dynamic light scattering (DLS) using Malvern Zetasizer (Nano ZEN3600, Malvern, UK). The zeta potential of polymeric micelles was also estimated using the same equipment.

#### 2.1.5. Measurement of the Critical Micellar Concentration (CMC) of Plain and GE11 Cisplatin Micelles

The CMC of the GE11 cisplatin micelles were estimated and compared to plain cisplatin micelles by DLS [36] using Malvern Zetasizer (Nano ZEN3600, Malvern, UK). For this purpose, plain and GE11 cisplatin micelles having polymer concentrations ranging from 1000 to 3 µg/mL were prepared. Briefly, from a stock solution of 1000 μg/mL micellar solution, different concentrations of micelles were prepared by serial dilution. The lowest prepared concentration was 3 μg/mL. The intensity of scattered light for each of concentrations was measured at a scattering angle of 173° at 25 °C. The average intensity of scattered light from three measurements was plotted against polymer concentration. The intersection of the two linear graphs in the sigmoidal curve, i.e., the onset of a rise in the intensity of scattered light, was defined as the CMC value.

#### 2.1.6. Measurement of Cisplatin Encapsulation

The Pt(II) content in the GE11 cisplatin micelles was determined by ion coupled plasma mass spectrometer (ICP-MS, Agilent Technologies, Tokyo, Japan). The ICP operated at a radiofrequency power of 1550 W, and the flow rate of argon carrier gas was 0.9–1.0 L/min. Pt(II) was monitored at *m*/*z* 195. A standard curve in the Pt(II) concentration range of 100, 50, 20, 10, and 1 ppb was generated using atomic absorption standard. Appropriate dilutions of the test samples were prepared in 1% nitric acid (HNO_3_). Data were acquired and processed by ICP-MS ChemStation (Agilent Technologies, Santa Clara, CA, USA). The encapsulation efficiency (EE) and drug loading (DL) were calculated using the following equations:

$$\;EE\;\left(\%\right)=\;\frac{the\;amount\;of\;encapsulated\;cisplatin\;\left(mg\right)}{the\;total\;feeding\;amount\;of\;cisplatin\;\left(mg\right)\;}\;\times 100\;$$

$$\;DL\;\left(\%\right)=\;\frac{the\;amount\;of\;encapsulated\;cisplatin\;\left(mg\right)\;}{the\;total\;amount\;of\;polymer\;\left(mg\right)}\;\times 100\;$$

#### 2.1.7. In Vitro Release Studies

The release of free cisplatin and its micellar formulations (plain and GE11 cisplatin micelles) was measured using equilibrium dialysis method in PBS (pH = 7) and acetate buffer saline (pH = 5). Briefly, free cisplatin, plain or GE11 cisplatin micelles (4 mL) containing 30 µg/mL cisplatin were placed into a dialysis bag (Spectrapor, MWCO 3500) in a beaker containing 500 mL PBS or acetate buffer saline. The release study was performed at 37 °C in a Julabo SW 22 shaking water bath (Seelbach, Germany). At selected time intervals, 100 μL samples were withdrawn from the inside of dialysis bag and replaced with fresh medium for ICP-MS analysis. The percent cumulative amount of cisplatin released was calculated and plotted as a function of time. The release profiles of plain and GE11 cisplatin micelles were compared using the similarity factor, *f_2_*, and the profiles were considered significantly different if *f_2_* < 50 [37]. The similarity factor, *f_2_,* was calculated using the following equation [38].

(1) $$\;f_{2}=50\;\times \log\left({\left[1+\left(\frac{1}{n}\right)\overset{n}{\underset{j=1}{\sum }}{\left|Rj-Tj\right|}^{2}\right]}^{-0.5}\times 100\right)\;$$

where *n* is the sampling number, *R*_j_ and *T*_j_ are the percents released of the reference and test formulations at each time point *j*.

#### 2.1.8. Cell Culture

MDA-MB-231 cells was obtained from ATCC (Manassas, VA, USA) and maintained in RPMI 1640 medium supplemented with 10% fetal bovine serum (Invitrogen, Karlsruhe, Germany), 100 units/mL penicillin, and 100 µg/mL streptomycin in a humidified incubator under 95% air and 5% CO_2_ at 37 °C. For hypoxic conditions, cells were cultured in a CO_2_ incubator maintained at 94% N_2_, 5% CO_2_, and 1% O_2_.

#### 2.1.9. Flow Cytometric Detection of Apoptosis using Annexin V-FITC and Propidium Iodide

Annexin V-FITC (Fluorescein IsoThioCyanate) and propidium iodide (PI) from BD Biosciences (FITC Annexin V Apoptosis Detection Kit I, #556547, BD Pharmingen™) was used to measure apoptotic cells by flow cytometry according to the manufacturer’s instructions. Briefly, both floating and adherent cells were harvested; adherent cells were collected by adding a warm solution of 10 mM ethylenediaminetetraacetic acid (EDTA) in PBS. The cells were centrifuged at 500 g for 5 min, washed with ice-cold 1 PBS twice and re-suspended in 400 µL binding buffer containing 5 µL Annexin V-FITC and 5 µL PI for 15 min at room temperature in the dark. Fluorescence was induced on a Beckman Coulter Cytomics Quanta SC MPL flow cytometer (10,000 events per sample). Spectral compensation was performed using Cell Lab Quanta analysis software (Cell Lab Quanta™ SC MPL, Beckman Coulter, Mississauga, ON, Canada). The number of viable and apoptotic cells were quantified by events in the quadrants. The results were expressed as the percentage of apoptotic cells at the early stage (PI negative and Annexin V positive, lower right quadrant), apoptotic cells at the late stage (PI positive and Annexin V positive, upper right quadrant), necrotic cells (PI positive and Annexin V negative, upper left quadrant) and viable cells (PI negative and Annexin V negative, lower left quadrant).

#### 2.1.10. MTT Assay

MDA-MB-231 cells (9 × 10^3^ cells/well) were seeded in 96-well plates overnight, and on the following day, they were exposed to increasing concentration of cisplatin (free drug/cisplatin micelles) and then incubated for 48 h under hypoxia or normoxia. For combination therapy, MDA-MB-231 cells (9 × 10^3^ cells/well) were seeded in 96-well plates overnight. On the following day, cells were treated with Tat-tagged form of cyclic peptide inhibitor of HIF-1 (*cyclo*-CLLFVY) named P1 (50 µM per well) [39] or Stattic (2 µM per well), or combination of P1 and Stattic for 4 h under normoxia, to give a final concentration of 2 µM and 50 µM per well, respectively. After 4 h incubation, cells were treated with cisplatin (50 µM) (as a free drug, plain cisplatin micelles or GE11 cisplatin micelles) and then incubated for additional 48 h under hypoxic or normoxic conditions. Cellular viability was assessed by the (3-(4,5-dimethylthiazol-2-yl)-2,5-diphenyltetrazolim bromide (MTT) assay. Briefly, MTT solution (5 mg/mL) was added to incubated cells for 4 h at 37 °C. Then the medium was replaced by DMSO to dissolve the crystals. Optical density was measured spectrophotometrically using a plate reader (Synergy H1 Hybrid Reader, Biotek, Winooski, VT, USA) at 570 nm. The cellular activity ratio was represented relative to control (untreated group, cells with media only).

#### 2.1.11. Western Blot

To measure the expression level of different proteins, MDA-MB-231 cells (2 × 10^5^ cells/well) were seeded in 6-well plates overnight. After treatment, cells were washed with cold 1X PBS and lysed using radioimmunoprecipitation assay buffer (RIPA lysis buffer) that was supplemented with 0.1 mM phenylmethylsulfonyl fluoride (PMSF) (Sigma-Aldrich), a protease Inhibitor Cocktail Set III, Animal-Free–Calbiochem (#535140, Millipore), and a phosphatase Inhibitor Cocktail Set II (#524625, Millipore). The lysate was then incubated on ice for 30 min, which was followed by centrifugation at 17,000× *g* for 20 min to remove genomic DNA. Protein quantification was made by the bicinchoninic acid (BCA) protein assay kit (Pierce, Rockford, IL, USA), and equal amounts of protein (35–40 μg) were loaded in 4−15% Tris-Glycine gradient gel (#456-1084, Biorad, Pleasanton, CA, USA). After gel electrophoresis, proteins were transferred to a nitrocellulose membrane. Membranes were probed with antibodies against phospho-STAT3 (Tyr705) (pSTAT3) (#9131, Cell Signaling Technologies, Danvers, MA, USA), Total-STAT3 (T-STAT3) (#8768s, Cell Signaling Technologies), EGFR (#2232, Cell Signaling Technologies), and glyceraldehyde 3-phosphate dehydrogenase (GAPDH) (#sc-47724, Santa Cruz Biotechnologies). Proteins were then detected using peroxidase-conjugated anti-mouse IgG (#7076, Cell Signaling Technologies) or anti-rabbit IgG (#7074, Cell Signaling Technologies) and visualized by enhanced chemiluminescence (Pierce ECL Western Blotting Substrate, #32106, Thermo Scientific, Rockford, IL, USA). Representative results of three independent Western blot analyses are shown in the Figure 4 and Figure 5, Figure S4 and Figure S5.

#### 2.1.12. Cisplatin Cellular Uptake

Cellular uptake of cisplatin was quantified by ICP-MS (Agilent Technologies, Tokyo, Japan). MDA-MB-231 cells (65 × 10^4^ cells/flask) were seeded in 25 cm^2^ flasks overnight. Cells were exposed to free cisplatin or its micellar formulations (plain and GE11 cisplatin micelles) (166 μM) for 24 h under normoxic and hypoxic conditions. On the following day, the medium was aspirated, cells were rinsed with cold PBS, detached using trypsin-EDTA, aliquoted in duplicate in 1.5 mL micro-centrifuge tubes and pelleted by centrifugation at 500× *g* for 5 min. One of each duplicate cell pellet was digested with 20% (*v*/*v*) HNO_3_ overnight at 60 °C and analyzed for Pt(II) content by ICP-MS. The other duplicate was lysed using RIPA lysis buffer that was supplemented with 0.1 mM phenylmethylsulfonyl fluoride (PMSF) (Sigma-Aldrich, St. Louis, MO, USA), a protease Inhibitor Cocktail Set III, Animal-Free–Calbiochem (#535140, Millipore), and a phosphatase Inhibitor Cocktail Set II (#524625, Millipore) and quantified for protein content using the BCA protein assay kit (Pierce, Rockford, IL, USA). The cell uptake was expressed as cisplatin/cell protein (μg)/μg).

#### 2.1.13. Statistical Analysis

The statistical analysis was performed by Graphpad Prism (version 5.00, Graphpad Software Inc., La Jolla, CA, USA). Statistical analysis was performed either using unpaired Student’s *t* test or one-way ANOVA (analysis of variance) with Tukey post-test analysis. Statistical significance is denoted by (*p* < 0.05). All graphs represent the average of at least three independent experiments with triplicates unless mentioned otherwise in the text, or graphs. Results were represented as the mean ± standard deviation (SD).

## 3. Results

### 3.1. Successful Synthesis of GE11 Conjugated Poly(ethylene oxide)-poly(α-carboxyl-ε-caprolactone)(PEO-PCCL) Block Copolymer and Its Self-assembly

GE11 showed a conjugation efficiency reaching 70% of an added peptide as quantified by reversed phase HPLC to the acetal-PEO-PCCL micellar surface (Figure S2). The molar conjugation percent for GE11 conjugated PEO-PCCL block polymer was ∼35%. In other words, for 100 mole block copolymers, there is around 35 mole conjugated peptide.

The peptide conjugation was also confirmed by ^1^H NMR (Figure S3). Signals from 6.5 to 8.5 ppm in ^1^H NMR spectra of GE11 peptide correspond to aromatic protons in its structure (*n* = 20). To calculate the molar conjugation percent for GE11-conjugated PEO-PCCL block polymer, first, the summation of integration of peaks from 6.5 to 8.5 ppm in acetal-PEO-PCCL spectra was subtracted from the summation of integration of peaks from 6.5 to 8.5 ppm in GE11-PEO-PCCL spectra. The obtained value corresponded to the integration of aromatic hydrogens of GE11 peptide (*n* = 20). The degree of conjugation of GE11 was then determined by calculating the peak intensity ratio of methylene protons of PCCL segment (OCH_2_CH_2_CH_2_CH_2_ ((COOH)CO): δ = 4.1 ppm) and the value calculated for the integration of aromatic hydrogens of GE11 peptide. The calculated degree of conjugation was 0.45 which corresponds to 45 mole peptide per 100 mole block copolymers (Figure S3). The calculated peptide conjugation degree by ^1^H NMR (0.45 mol/mol) was slightly higher than what was calculated by HPLC (0.35 mol/mol).

As summarized in Table 1, both plain and GE11 cisplatin micelles showed similar average diameters around 80 nm with a low polydispersity index. Critical micellar concentration (CMC), cisplatin encapsulation efficiency and drug loading of both micelles were also comparable. Overall, both micellar formulations showed similar characteristics, and it appeared as though the presence of the GE11 peptide did not alter different micellar properties (*p* > 0.05, Student’s *t* test).

**Figure 1:**
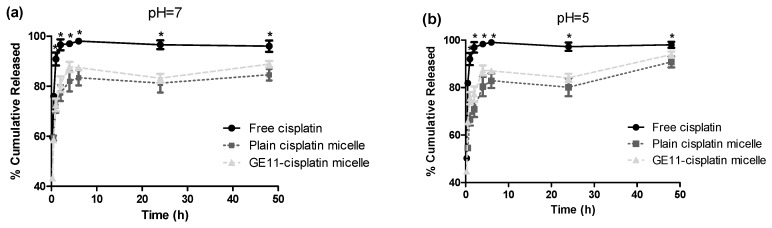
Percent cumulative release profile of cisplatin from plain and GE11 cisplatin micelles at different pHs in (**a**) PBS (pH = 7.4) and (**b**) acetate buffer saline (pH = 5.0). (*) denotes where the cumulative release of free drug appeared to be significantly different from plain and GE11 cisplatin micelles at the related time points (*p* < 0.05, Student’s *t* test). Data are represented as mean ± SD (*n* = 3).

The in-vitro release of cisplatin from both micelles was investigated in phosphate buffered saline (PBS) (pH = 7.4), and acetate buffered saline (pH = 5.0) using a dialysis method. Both micellar formulations showed burst release at the early time points (<1 h). The cumulative drug release appeared to be significantly slower at the later time points (>2 h) as compared to free drug for both micelles, however (Student’s *t* test, *p* < 0.05). As shown in Figure 1a, micellar formulations showed ∼60% release of cisplatin within 30 min in PBS as compared to ∼80% release of free drug in the same media. After 48 h, in PBS, ∼85% of the incorporated drug was released from micelles to media, compared to complete 100% release for free drug. Similar results were obtained for micellar formulations of cisplatin in acetate buffered saline (pH = 5.0) (Figure 1b) where a significant reduced drug release was achieved at later time points (>2 h) (Student’s *t* test, *p* < 0.05) for micellar cisplatin compared to free drug. No difference was observed between the release profiles of plain versus GE11 cisplatin micelles in either media (*f*_2_ > 50). Of note, in acetate buffered saline (pH = 5) complete release of drug after 48 h was seen, whereas only 80% drug was released in PBS at the same time point (pH = 7.4) (Figure 1b), although the overall profile of drug release did not show a significant difference between the two pHs (*f2* > 50).

### 3.2. Hypoxia Induces Chemoresistance to Free Cisplatin in MDA-MB-231 Cells

**Figure 2:**
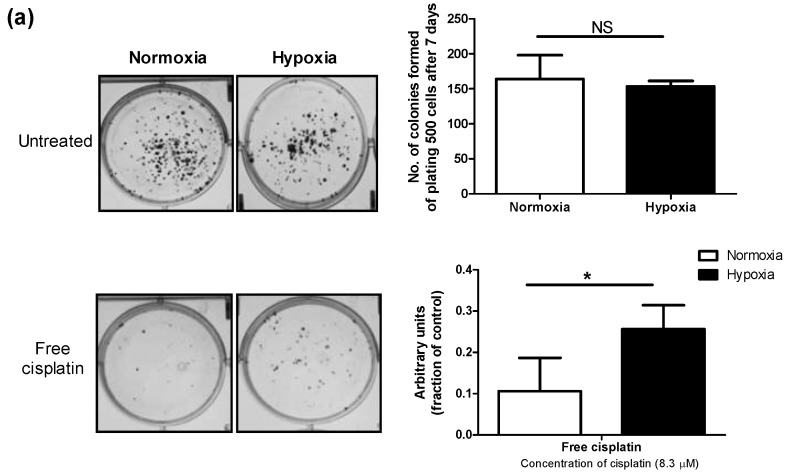

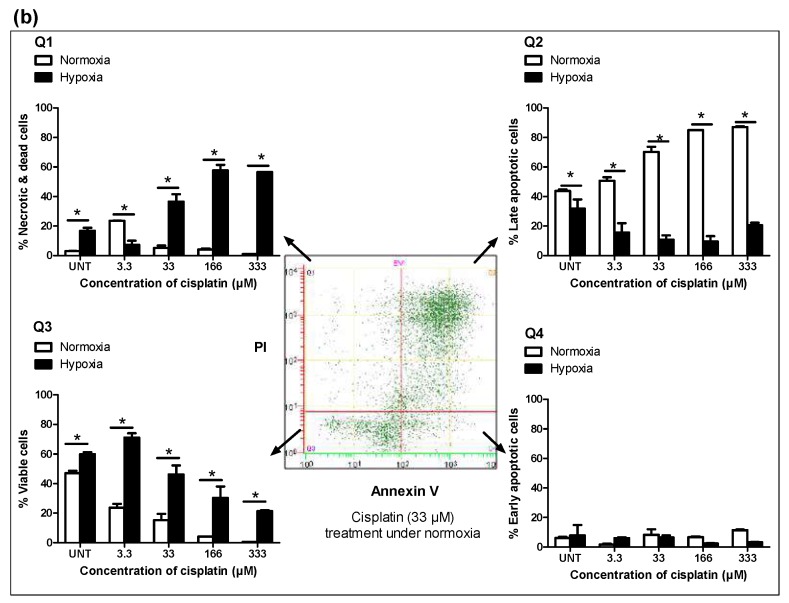
Hypoxia confers chemoresistance to free cisplatin in MDA-MB-231 cells. (**a**) Colony formation ability was assessed for cells treated with cisplatin (8.3 µM) after 24 h incubation under normoxic or hypoxic conditions. Cells were then re-plated at a density of 500 cells/well in duplicate in six-well plates under normoxia. The number of colonies formed from 500 cells after 7 days was graphed. (**b**) Cisplatin induced-apoptosis under normoxic and hypoxic conditions was measured by flow cytometric analysis of Fluorescein IsoThioCyanate (FITC) Annexin V staining in a buffer containing propidium iodide. MDA-MB 231 cells were left untreated or treated with increasing concentrations of cisplatin for 48 h. Flow cytometry analysis showed different populations of (Q1) necrotic or already dead cells (PI positive), (Q2) cells in end-stage apoptosis (FITC Annexin V and PI positive), (Q3) viable cells (FITC Annexin V and PI negative), and (Q4) cells in early stage of apoptosis (FITC Annexin V positive and PI negative). 2D plot is representative of cells treated with cisplatin (33 µM) under normoxia for 48 h. Data are represented as mean ± SD (*n* = 3). (*) denotes a significant difference between hypoxic and normoxic groups at each individual concentration (Student’s *t* test, *p* < 0.05).

**Figure 3:**
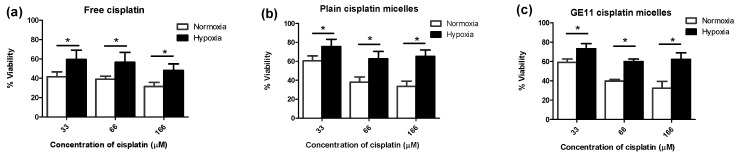
Hypoxia confers chemoresistance to free and micellar formulations of cisplatin in MDA-MB-231 cells. Viability of MDA-MB-231 cells was measured by MTT assay for cells treated with increasing concentrations of (**a**) free cisplatin; (**b**) plain cisplatin micelles and (**c**) GE11 cisplatin micelles under hypoxic or normoxic conditions for 48 h. (*) denotes a significant difference between groups at each individual concentration (Student’s *t* test, *p* < 0.05).

The MDA-MB-231 cells incubated under hypoxic conditions were shown to be less sensitive to cytotoxic effects of cisplatin as measured by MTT assay. Cells cultured under hypoxia had a significantly higher number of colonies surviving cisplatin treatment than cells grown under normoxic conditions, as well (Figure 2a). Moreover, apoptosis induced by cisplatin was significantly reduced under hypoxia, as evidenced by a significant decrease in the proportion of late apoptotic cells measured by FITC Annexin V/propidium iodide (PI) assay (Figure 2b). The viable proportion of cells also showed a significant increase under hypoxic conditions compared to normoxic ones, when treated with cisplatin (Figure 2b). Interestingly, the proportion of necrotic cells following treatment with cisplatin under hypoxic conditions increased compared to normoxia, pointing to a change in the predominant mode of cisplatin-induced cell death under hypoxic conditions. This phenomenon was also previously reported for prostate carcinoma [40]. Similar to observation on free cisplatin (Figure 3a), we found less sensitivity towards polymeric micellar cisplatin in MDA-MB-231 cells cultured under hypoxic conditions by MTT assay (Figure 3b,c). There was also no significant difference between cell responses to micellar versus free cisplatin irrespective of the oxygen content for cell culture (one-way ANOVA with Tukey post-test, *p* > 0.05).

### 3.3. Modification of Cisplatin Micelles with GE11 Peptide Enhances the Cellular Uptake of Cisplatin, but Does Not Affect its Cytotoxicity in MDA-MB-231 Cells

Modification of polymeric micelles with EGFR targeting peptide, GE11, was not able to increase the cytotoxicity of incorporated cisplatin towards MDA-MB-231 cells under normoxic or hypoxic conditions (Figure 3c versus 3b).

**Figure 4:**
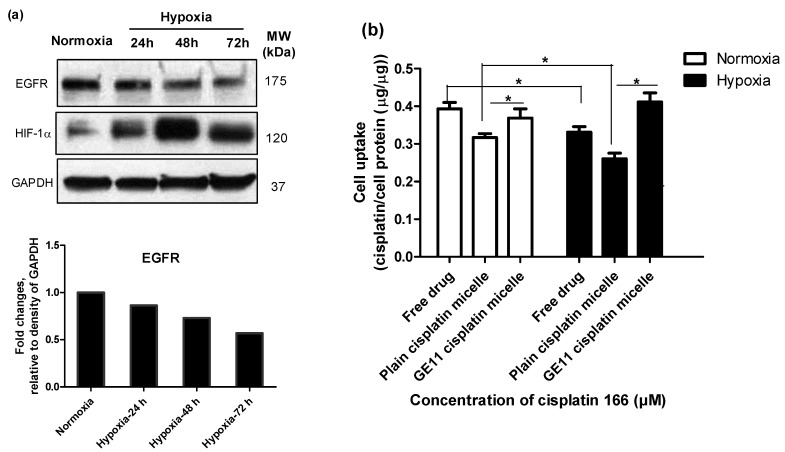
Modification of cisplatin micelles with GE11 peptide enhances the cellular uptake of cisplatin in MDA-MB-231 cells. (**a**) High levels of epidermal growth factor receptor (EGFR) expression under normoxia and hypoxia in MDA-MB-231 cells; (**b**) The GE11-peptide decoration of cisplatin micelles enhanced cellular uptake of cisplatin under hypoxia in MDA-MB-231 cells and bridged the gap of its cellular uptake under hypoxia and normoxia. Cisplatin content was measured by ion coupled plasma mass spectrometer (ICP-MS) after 24 h treatment of cells with cisplatin (166 µM) under hypoxia or normoxia. (*) denotes a significant difference between compared groups (Student’s t test, *p* < 0.05).

This was despite high levels of EGFR expression by MDA-MB-231 cells under normoxic or hypoxic conditions (Figure 4a) [17], that led to significantly higher cisplatin uptake following modification of polymeric micelles with GE11 peptide compared to plain micelles under both conditions (Figure 4b).

As shown in Figure 4a, the levels of EGFR expression under hypoxia were time-dependent. At the 24-h time point, there was no significant difference between the expression of EGFR under hypoxia and normoxia. However, the expression of EGFR was reduced under hypoxia for longer incubation times. Thus, for the purpose of comparability, we chose the 24-h time point for performing the cell uptake studies. When we treated the cells with the low concentration of cisplatin, due to multiple steps of digestion and washing for a sample preparation, we could not quantify the (low) amount of intracellular cisplatin. To compensate for the limitation of the measurement method, we chose to treat the cells with a high concentration of cisplatin (166 µM). As shown in Figure 4b, intracellular levels of cisplatin were found to be significantly reduced under hypoxia for the free drug (∼1.6-fold decrease) as well as plain cisplatin micelles (∼1.4-fold decrease). Nonetheless, the presence of the GE11 peptide on micelles appeared to compensate for the hypoxia-mediated reduction in cellular cisplatin levels. GE11 modified micelles showed similar intracellular drug levels under both hypoxic and normoxic conditions.

### 3.4. Co-Treatment with Pharmacological Inhibitors of HIF-1 and STAT3 Potentiates the Anticancer Activity of Free Cisplatin, as well as Its Micellar Formulations in Hypoxic MDA-MB-231 Cells

**Figure 5:**
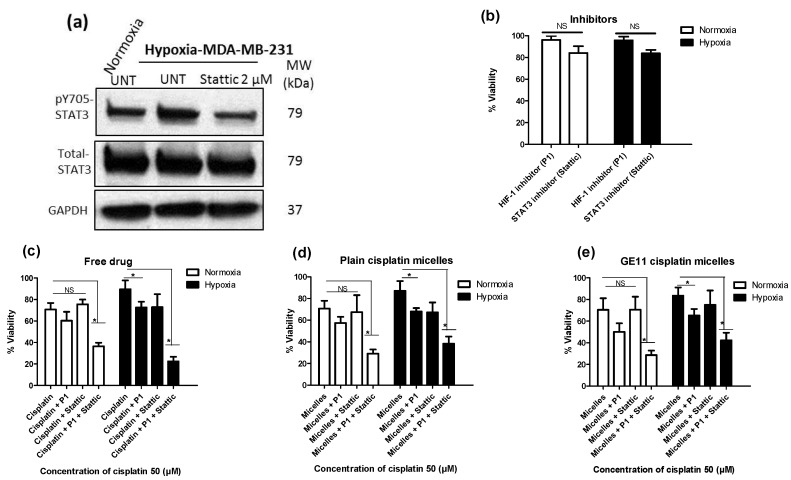
Dual pharmacological inhibition of signal transducer and activator of transcription 3 (STAT3) and hypoxia inducing factor-1 (HIF-1) in combination with free cisplatin or its micellar formulations successfully reversed hypoxia-induced chemoresistance. (**a**) Lower expression of pSTAT3 in MDA-MB-231 cells after treatment with STAT3 inhibitor (Stattic). Phosphorylation of STAT3 Tyr705 was analyzed by Western blot. (**b**–**d**) Viability of MDA-MB-231 cells was measured by MTT assay for cells which first pre-incubated with the HIF-1 inhibitor (named P1) (50 µM), the STAT3 inhibitor (Stattic) (2 µM) or both under normoxia for 4 h and then incubated under hypoxia for additional 48 h (b) in the absence of cisplatin or in the presence of 50 μM (c) free drug; (d) plain cisplatin micelles and (**e**) GE11 cisplatin micelles. (*) denotes a significant difference between compared groups (one-way ANOVA with Tukey post-test, *p* < 0.05).

We have previously reported on the role of STAT3 up-regulation under hypoxia as a mediator of hypoxia-induced resistance to cisplatin in MDA-MB-231 cells [30,41]. Here, we assessed the chemo-sensitizing effect of pharmacological inhibitors of STAT3 and/or HIF-1 (a known mediator of hypoxia- induced chemoresistance) in MDA-MB-231 cells treated with different formulations of cisplatin. For this purpose, a cyclic peptide that inhibits the assembly and function of the HIF-1 transcription factor (*cyclo*-CLLFVY) [39], as well as a known inhibitor of STAT3, i.e., Stattic, [42] were used in combination with free, plain and GE11 cisplatin micelles and the cytotoxicity of cisplatin against MDA-MB-231 cells was measured using MTT assay under normoxic and hypoxic conditions. The tat-tagged form of *cyclo*-CLLFVY (named P1), a cyclic peptide which has shown to prevent the dimerization of HIF-1α/HIF-1β complex by binding to HIF-1α and subsequently inhibit HIF-1 mediated hypoxia response [39,43], was used as a HIF-1 inhibitor. Stattic, a small molecule shown to inhibit the dimerization and activation of STAT3 mainly through prevention of its phosphorylation [42], was used as a STAT3 inhibitor. As shown in Figure 5a and Figure S4, successful inhibition of phosphorylation of STAT3 was achieved in MDA-MB-231 cells using Stattic (2 μM). Both P1 and Stattic showed minimal non-specific cytotoxicity at their respective effective dose for the inhibition of HIF-1 and STAT3 (50 and 2 μM, respectively) as shown by MTT assay (Figure 5b).

Under normoxic conditions, co-treatment of MDA-MB-231 cells with P1 and cisplatin formulations showed a trend towards potentiating the anticancer effect of cisplatin, plain cisplatin micelles or GE11 cisplatin micelles, as measured by MTT assay, although the difference was not statistically significant (Figure 5c–e, white bars). However, under hypoxic conditions, combination of P1 with free cisplatin (Figure 5c, black bars), plain cisplatin micelles (Figure 5d, black bars) and GE11 cisplatin micelles (Figure 5e, black bars) significantly enhanced the cytotoxicity of cisplatin as part of each formulation (one-way ANOVA with Tukey post-test, *p* < 0.05). This was not observed when different formulations of cisplatin were combined with the inhibitor of STAT3, Stattic, under normoxic conditions (Figure 5c–e, white bars). Under hypoxia, a combination of Stattic with free cisplatin (Figure 5c, black bars), plain cisplatin micelles (Figure 5d, black bars) and GE11 cisplatin micelles (Figure 5e, black bars) showed a trend towards increasing the cytotoxicity of cisplatin, although the results were not statistically significant.

Simultaneous co-treatment of cells with inhibitors of HIF-1 and STAT3 enhanced the cytotoxic effects of free cisplatin (Figure 5c), plain cisplatin micelles (Figure 5d) and GE11 cisplatin micelles (Figure 5e) in both normoxic and hypoxic MDA-MB-231 cells (one-way ANOVA with Tukey post-test, *p* < 0.05). Simultaneous co-treatment of cells with inhibitors of HIF-1 and STAT3 was the most effective approach in the reversal of HICR for free cisplatin as well its nano-formulations in this study. Co-treatment of cells with inhibitors of HIF-1 and STAT3 increased cisplatin toxicity under both conditions for all different drug formulations; the enhancement was more noticeable under hypoxia. For example, there was a ∼3.51-fold increase of cytotoxicity under hypoxia versus ∼1.93 under normoxia for free drug, ∼2.42-fold increase of cytotoxicity under hypoxia versus ∼2.27 under normoxia for plain cisplatin micelles, and ∼2.46-fold increase of cytotoxicity under hypoxia versus ∼1.97 under normoxia for GE11 cisplatin micelles. All the comparisons were made to cells treated with the related formulation in the absence of the inhibitors. Overall, free drug showed the highest cytotoxicity enhancement (∼3.51-fold increase) after co-treatment of cells with the inhibitor of HIF-1 and STAT3, following by GE11 cisplatin micelles (∼2.46-fold increase) and plain cisplatin micelles (∼2.42-fold increase) under hypoxia; whereas the cytotoxicity enhancement was similar for all the formulations under normoxia (∼2.05-fold increase).

In all of the above experiments, irrespective of the oxygen pressure under which the cells were cultured, no significant difference between the cytotoxicity of free drug and micellar formulations was observed. In addition, GE11 cisplatin micelles showed a similar profile of toxicity as compared to plain cisplatin micelles in MDA-MB-231 cells.

## 4. Discussion

Hypoxia is widely known to be associated with chemoresistance in different types of solid tumors [2,3] including TNBC [4,5]. The increase in the expression of HIF-1α has long been associated with the development of chemoresistance [4]. More recently, an increase in the activation of STAT3 following hypoxia is shown to be at least partly responsible for mediating chemoresistance in the human ovarian cancer (i.e., A270 cells) and TNBC (i.e., MDA-MB-231 cells) [3,30]. Overexpression of epidermal growth factor receptor (EGFR) and tumor hypoxia have also been shown to correlate with worse outcomes in several types of cancers including breast cancer [16,44]. This coexistence of hypoxia and EGFR may implicate a survival advantage of hypoxic cells that also express EGFR.

Design and development of nanotechnology products as a means to enhance the therapeutic index of anticancer drugs has been explored intensely in the past few decades. In successful cases, nano-formulations of anticancer drugs have shown to extend their blood circulation time leading to improved accumulation of the drug in solid tumors mostly by the enhanced permeability and retention (EPR) effect and/or decrease drug exposure and toxicity to normal tissues [9,45]. However, owing to their nanoscopic size and slow drug release, nano-formulations of the anticancer drugs are also speculated to provide limited access of the drug to hypoxic cancer cells that are located in areas of tumor away from blood vessels [46].

The objective of this study was first to evaluate the cytotoxic behavior of a newly developed nano-formulations of cisplatin on hypoxia-induced chemoresistant (HICR) MDA-MB-231 cells, a TNBC cell line; and secondly to explore feasible approaches for overcoming HICR against free and nano-formulations of cisplatin. For the latter purpose, we developed EGFR-targeted cisplatin micelles using an EGFR-ligand, GE11 peptide and investigated the effect of combination therapy with inhibitors of HIF-1 and STAT3 as the key mediators of HICR in this cell line.

Similar to plain cisplatin micelles, the GE11 cisplatin micelles showed slightly accelerated drug release in an acidic environment (Figure 1b). The ability of acid-triggered release of these micelles is important; tumors, particularly those with hypoxic regions, usually have lower extracellular pH than that of normal tissues [47]. In this case, the acid-triggered release of cisplatin from the micellar formulations may encourage drug release from the nano-formulation in the hypoxic tumor. This may compensate for the restrictions faced by these nano-formulations for penetrating the tumor core. It is of note that this feature, i.e., the acid-triggered release of cisplatin from its micellar formulations, was not investigated in our cell cytotoxicity studies as the pH of the cell culture media was maintained at 7.4.

In concert with our previous study [30], we found that hypoxia significantly induced resistance against free cisplatin in MDA-MB-231 cells (Figure 2 and Figure 3a). MDA-MB-231 cells also showed similar resistance against nano-formulations of cisplatin under hypoxia (Figure 3b,c). GE11 cisplatin micelles showed increased intra-cellular levels of cisplatin compared to free and plain cisplatin micelles under hypoxia, reaching intracellular cisplatin levels similar to that of the free drug under normoxic conditions (Figure 4b). EGF receptors (EGFR) are internalizing receptors [48] with GE11 peptide as their ligand [29]. Thus, surface modification of cisplatin micelles with the GE11 peptide in this study was made to compensate for lower uptake of cisplatin under hypoxic conditions in MDA-MB-231 cells expressing high levels of EGFR. The upregulation of ATP-binding cassette (ABC) drug transporters, particularly ABCC2 and ABCC6, has been shown as one of the possible mechanisms responsible for active efflux of free cisplatin under hypoxia in MDA-MB-231 cells, which subsequently contributes to a decrease in the cellular levels of free cisplatin in this cell line [30]. Thus, the GE11 modified micelles have a potential to bypass ABC-transporter mediated drug efflux.

In spite of enhanced intracellular cisplatin levels by the GE11 modified micelles, cytotoxicity of cisplatin as part of GE11 micelles was not improved over plain cisplatin micelles, and both formulations showed similar cytotoxicity in MDA-MB-231 cells under normoxic and hypoxic conditions. In addition, irrespective of the oxygen pressure under which the cells were cultured, no significant difference between the cytotoxicity of free drug and micellar formulations was observed. We are speculating two reasons for this observation: (a) release of cisplatin from micelles at > 24 h incubation of micelles with the cells have contributed to the similar profile of toxicity between free drug and micellar formulations; (b) the increased level of cell uptake for cisplatin by GE11 cisplatin micelles is not enough to pass the threshold required for bypassing the mechanisms of cisplatin resistance. More thorough studies are required to elucidate the reason, which will be the subject of our future investigations.

The master regulator of cellular adaptation under hypoxia is believed to be the hypoxia-inducible factor (HIF) protein [47,49]. HIF is a heterodimeric transcription factor comprised of an oxygen-regulated unit, HIF-1α, as well as a constitutively expressed beta unit, HIF-1β. In the presence of oxygen, once HIF-1α is produced; it will be hydroxylated, ubiquitinated, and degraded. In contrast, in the absence of oxygen, HIF-1α is stabilized, dimerizes with HIF-1β, and the HIF heterodimers translocate to the nucleus where it activates the transcription of various downstream targets, many of which are known to be involved in cancer aggressiveness and chemoresistance [47,49,50]. The HIF-1 inhibitor used in our study (P1) specifically inhibits HIF-1α dimerization with HIF-1β and subsequently inhibits HIF-1 transcription factor activity [39]. Our results showed that the combination of P1 with cisplatin significantly enhanced cisplatin toxicity under hypoxia; however, it was not effective under normoxic conditions. Co-treatment of cells with the HIF-1 inhibitor resulted in similarly enhanced cytotoxicity for all cisplatin formulations under hypoxia as compared to cells treated with the related formulation in the absence of inhibitor. The effectiveness of combination therapy under hypoxia is likely due to the higher expression of HIF-1α under hypoxia as compared to normoxic conditions.

When combining a STAT3 inhibitor (Stattic) with cisplatin treatment, we did not observe any significant changes in the profile of cytotoxicity of cisplatin in MDA-MB-231 cells. This was unexpected given the results of previous studies supporting a role for activation of STAT3 in conferring HICR to cisplatin [3,30], and the effectiveness of STAT3 siRNA in sensitization of MDA-MB-231 cells to cisplatin. The exact reason behind the discrepancy is not clear and warrants further investigation. The efficiency of inhibition of STAT3 activation may be varied following siRNA transfection versus Stattic treatment at applied doses. The difference in the expression levels of pSTAT3 and its downstream targets involved in aggressiveness and chemoresistance of cancer cells (e.g., c-Myc [51]) following STAT3 siRNA versus Stattic treatment is speculated to have a role in this observation (Figure S5).

It should be noted that some cancer cells, including MDA-MB-231 cells, constitutively express high levels of HIF-1α [52] and pSTAT3 [53] under normal oxygen conditions, although their levels of expression are significantly lower as compared to hypoxia. The inhibition of HIF-1 or STAT3, alone, was not effective in enhancing the cytotoxic effect of cisplatin in our study under normoxic conditions. However, when HIF-1 and STAT3 both were inhibited, the cytotoxic effects of cisplatin increased under both normoxic and hypoxic conditions. 

Previous studies have also provided support for the combined targeting of HIF-1 and STAT3 under hypoxia for enhancing anti-tumor activity. For instance, administration of a series of dual inhibitors of HIF-1α and STAT3 (in the absence of anticancer agent) resulted in significant anti-proliferative activity across a panel of various cancer cell lines [54]. Furthermore, it has been shown that the combination of other pharmacological inhibitors of HIF-1α and STAT3 enhanced prostate tumor growth suppression [55]. The results of our study, however, provide proof-of-principle for the use of HIF-1 and STAT3 inhibitors (individually or in combination) for sensitization of resistant cells under hypoxia to cisplatin and its micellar formulations in TNBC. Our present efforts for combination therapy are made by separate addition of inhibitors and cisplatin micelles in-vitro. Addition of inhibitors to the cell culture medium was done a few hours before the treatment with cisplatin or its micellar formulations. This approach was expected to provide enough time for the inhibitors to execute their inhibitory effect and sensitize the cells to the treatment. However, moving forward to in-vivo studies, delivery of both inhibitors and cisplatin within the same or separate micellar formulation can be explored. The use of nanodelivery systems for drugs is expected to provide a control over the time and extent of their delivery in solid tumors, maximizing their benefit

## 5. Conclusions

In summary, we have shown that the modification of cisplatin micelles with EGFR ligand (i.e., GE11 peptide) compensated for the hypoxia-mediated reduced cisplatin uptake, although this approach was not successful in increasing the levels of cytotoxicity of the drug. Importantly, our findings suggest that the potency of conventional (i.e., cisplatin) and nano-formulations (i.e., cisplatin micelles) can be enhanced under hypoxia once inhibitors of major cellular and molecular players of hypoxia-induced chemoresistance (i.e., HIF-1 and STAT3) were used in combination. To conclude, we have provided evidence to support that the rational therapeutic drug combination of sensitizing drugs with other therapies should be used to overcome drug resistance.

## Figures and Tables

**Table 1:** Characteristics of Cisplatin and GE11 Cisplatin Micelles (*n* = 3).

Micelle ^a^	Average Diameter ± SD (nm) ^b^	PDI ± SD ^c^	Zeta potential ± SD (mV)	CMC ± SD (μg/mL) ^d^	EE ± SD (%) ^e^	DL ± SD (%) ^f^	Drug/polymer ± SD (mol/mol)
Cisplatin plain micelle	84.4 ± 2.6	0.263 ± 0.11	−13.3 ± 1.2	65.1 ± 5.5	12.4 ± 0.99	12.0 ± 1.41	3.93 ± 0.31
GE11 cisplatin micelle	84.1 ± 3.2	0.235 ± 0.18	−13.6 ± 0.95	70.5 ± 7.2	13.0 ± 2.95	15.5 ± 3.53	4.01 ± 0.93

^a^ Plain and GE11 cisplatin micelles consist of PEO_6000_-PCCL_3000_ block copolymers. The number shown in the subscript indicates average number molecular weight of each block determined by ^1^H NMR spectroscopy. ^b^ Z average measured by dynamic light scattering (DLS). ^c^ Average polydispersity index (PDI) of micellar size distribution measured by DLS. ^d^ Measured from the onset of rise in the intensity values of scattered light as a function of concentration of micelles by DLS. ^e^ Encapsulation efficiency $\left(\%\right)=\;\frac{the\;amount\;of\;encapsulated\;cisplatin\;\left(mg\right)}{the\;total\;feeding\;amount\;of\;cisplatin\;\left(mg\right)\;}\;\times 100$. ^f^ Drug loading $\left(\%\right)=\;\frac{the\;amount\;of\;encapsulated\;cisplatin\;\left(mg\right)}{the\;total\;amount\;of\;polymer\;\left(mg\right)}\;\times 100$.

## Supplementary Materials

The following are available online at http://www.mdpi.com/1999-4923/10/4/196/s1, **Figure S1.** (a) GE11 peptide structure and (b) MALDI-MS spectrum of GE11 peptide, **Figure S2.** Monitoring GE11 peptide conjugation to acetal-PEO-PCCL polymers using HPLC with UV detection at 214 nm, **Figure S3.** The conjugation of GE11 peptide onto acetal-PEO-PCCL was confirmed by 1H-NMR spectra of block copolymer before and after peptide conjugation. Samples (3–5 mg/mL) of acetal-PEO-PCCL and GE11-PEO-PCC were prepared in DMSO for 1H NMR analysis, **Figure S4.** Bar graph illustrating quantification of the Western blot densitometry analysis for Figure 5A. Densitometry data are expressed as fold changes compared to untreated normoxic group, normalized to GAPDH band intensity, **Figure S5.** The chosen experimental model for inhibition of STAT3 activation, STAT3 siRNA transfection versus pharmacological inhibition by Stattic, may result in different levels of expression of STAT3 and/or its downstream targets such as c-Myc protein. The differential levels of protein expression in MDA-MB-231 cells after transfection with STAT3 siRNA or treatment with Stattic (2µM) for 24 to 48 hours under hypoxia is depicted by Western blot.
