# Supplementary material for: Modulation of Hypoxia-Induced Chemoresistance to Polymeric Micellar Cisplatin: The Effect of Ligand Modification of Micellar Carrier Versus Inhibition of the Mediators of Drug Resistance
Source: Pharmaceutics. 2018 Oct 21;10(4):196. doi: 10.3390/pharmaceutics10040196 (PMC6320993; doi:10.3390/pharmaceutics10040196)
Supplement: Supplementary file 1 [file pharmaceutics-10-00196-s001.pdf]

Modulation of Hypoxia-Induced Chemoresistance to Polymeric Micellar Cisplatin: The Effect of Ligand Modification of Micellar Carrier Versus Inhibition of the Mediators of Drug Resistance

Hoda Soleymani Abyaneh[1], Amir Hassan Soleimani[1], Mohammad Reza Vakili[1], Rania Soudy[1,3], Kamaljit Kaur[1,4], Francesco Cuda[5], Ali Tavassoli[5], Afsaneh Lavasanifar[1,2] *

[1] Faculty of Pharmacy and Pharmaceutical Sciences, University of Alberta, Edmonton, AB T6G 2E1, Canada; hoda1@ualberta.ca (H.S.A.); asoleimani212@gmail.com (A.H.S.); vakili@ualberta.ca (M.R.V.); soudy@ualberta.ca (R.S.); kkaur@chapman.edu (K.K.); afsaneh@ualberta.ca (A.L.)

[2] Department of Chemical & Materials Engineering, Faculty of Engineering, University of Alberta, Edmonton, AB T6G 1H9, Canada

[3] Faculty of Pharmacy, Cairo University, Kasr El-Aini, Cairo 11562, Egypt

[4] School of Pharmacy, Chapman University, Irvine, California 92618, USA

[5] School of Chemistry, University of Southampton, Southampton SO17 1BJ, UK; francesco_cuda@yahoo.co.uk (F.C.); A.Tavassoli@soton.ac.uk (A.T.)

** Correspondence: Afsaneh Lavasanifar (A.L.), Email: afsaneh@ualberta.ca; Tel.: +1-780-492-27-42; fax: +1 780-492-12-17*

Supporting material

(a)

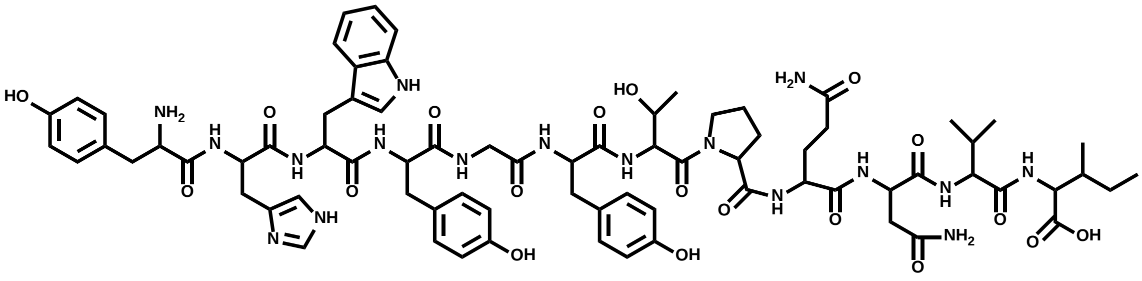

(b)

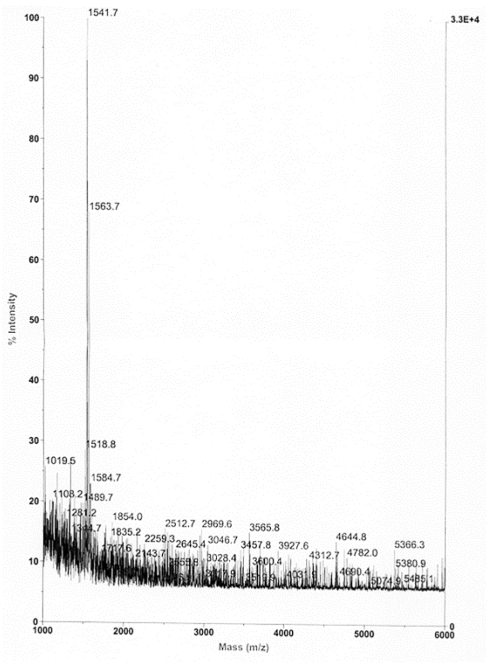

Figure S1. (a) GE11 peptide structure and **(b)** MALDI-MS spectrum of GE11 peptide**.**

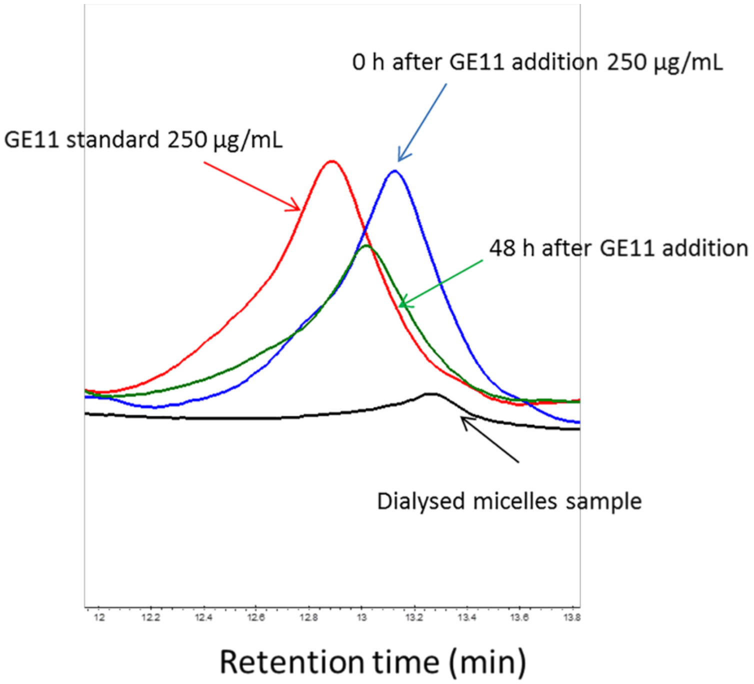

Figure S2. Monitoring GE11 peptide conjugation to acetal-PEO-PCCL polymers using HPLC with UV detection at 214 nm.

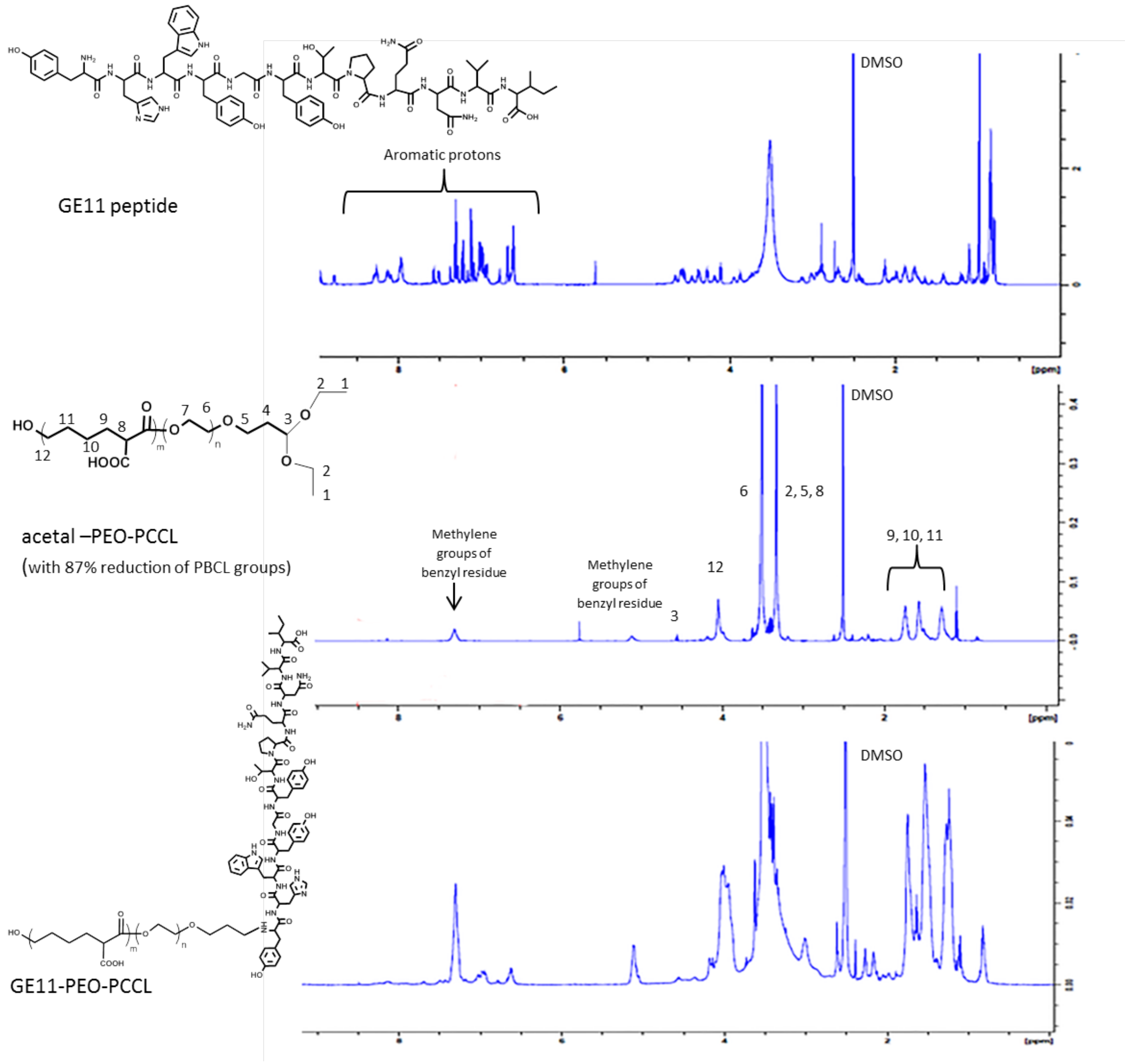

Figure S3. The conjugation of GE11 peptide onto acetal-PEO-PCCL was confirmed by ^{1}H-NMR spectra of block copolymer before and after peptide conjugation. Samples (3-5 mg/mL) of acetal-PEO-PCCL and GE11-PEO-PCC were prepared in DMSO for ^{1}H NMR analysis.

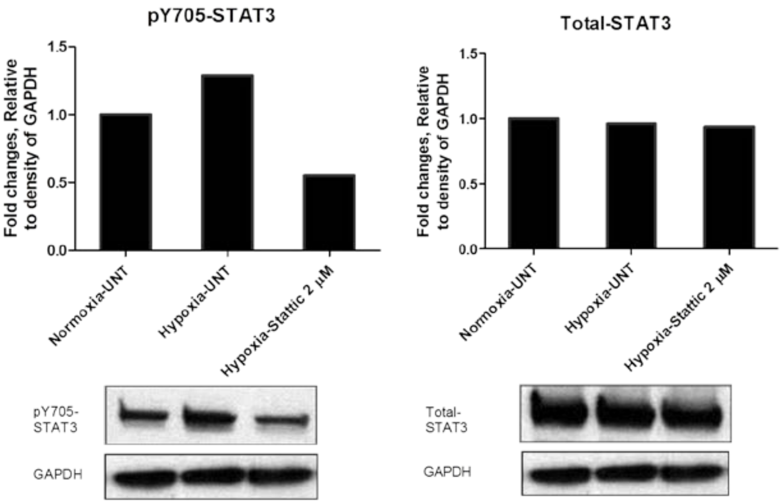

Figure S4. Bar graph illustrating quantification of the Western blot densitometry analysis for Figure 5A. Densitometry data are expressed as fold changes compared to untreated normoxic group, normalized to GAPDH band intensity.

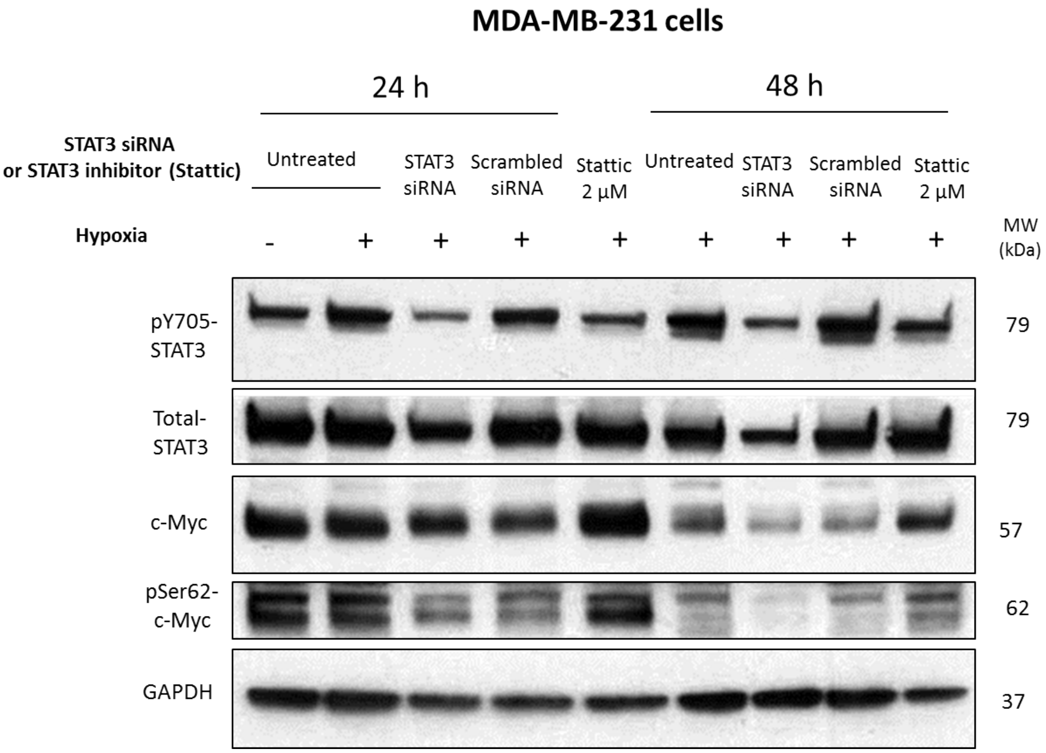

Figure S5. The chosen experimental model for inhibition of STAT3 activation, STAT3 siRNA transfection versus pharmacological inhibition by Stattic, may result in different levels of expression of STAT3 and/or its downstream targets such as c-Myc protein. The differential levels of protein expression in MDA-MB-231 cells after transfection with STAT3 siRNA or treatment with Stattic (2μM) for 24 to 48 hours under hypoxia is depicted by Western blot.
